# Attitudes and participation in fall risk management: A tripartite, multicenter cross-sectional study of physicians, nurses, and patients

**DOI:** 10.1371/journal.pone.0343136

**Published:** 2026-03-27

**Authors:** Qianqian Mou, Min You, Lin Tao, Junying Li, Yan Jiang, Xiaolian Jiang

**Affiliations:** 1 West China School of Nursing, Sichuan University, Chengdu, China; 2 Clinical Trial Center, West China Hospital, Sichuan University, Chengdu, China; 3 Department of Medical Oncology, Cancer Center, Cancer Day-Care Unit, West China Hospital, Sichuan University, Chengdu, China; 4 Department of Thoracic Oncology, West China Hospital, Sichuan University, Chengdu, China; 5 Nursing Department, West China Hospital, Sichuan University, Chengdu, China; University of Verona, ITALY

## Abstract

**Objective:**

This study aims to explore the attitudes and involvement of physicians, nurses and patients in fall risk management, focusing on the factors influencing physicians’ participation.

**Methods:**

This study utilized a convenience sampling, 4,272 participants (580 physicians, 2,775 nurses, 917 patients) from 19 Chinese provinces were enrolled via Questionnaire Star from 11 April to 31 May, 2024. The survey included general information, tripartite fall management perspectives, and 27-item medical staff/patient questionnaires. Medical staff evaluated fall risk through assessment, prevention, and management; patients reported adherence and staff participation.

**Results:**

Physicians and nurses showed comparable attitude scores (55.02 ± 8.124 vs 54.58 ± 9.096, *P* = 0.227), but physicians had higher participation (51.47 ± 9.703 vs 42.77 ± 12.052, *P* < 0.001). Key factors influencing physicians’ engagement included education level, gender, and nurses’ invitation frequency/awareness of fall management importance. Staff identified patient noncompliance, disease complexity, and low risk awareness as challenges; patients cited disease severity, environmental hazards, and insufficient education.

**Conclusion:**

Effective fall prevention requires multidisciplinary collaboration. Nurses predominantly lead fall management, while physicians demonstrate limited involvement, closely tied to nurses’ proactive engagement invitations. Cultivating a culture where nurses actively invite physician collaboration is critical to enhancing safety strategies.

## 1. Introduction

Patient safety is a critical concern in healthcare, with patient falls representing a serious threat to both physical and mental health, as well as quality of life, while imposing a substantial burden on public health systems [1,2]. According to the Global Report on Falls Prevention in Older Adults, falls are the leading cause of pain, disability, functional limitations, and even mortality among older adults [3]. The World Health Organization (WHO) reports that annually, 28% to 35% of individuals aged 65 and older experience at least one fall, with the prevalence increasing to 32% to 42% among those aged 70 and above [4]. Globally, approximately 37.3 million people require medical attention due to fall-related injuries each year, with over 680,000 fatalities, predominantly in low and middle-income countries [5]. In China, the age-standardized incidence rate (ASIR) and age-standardized mortality rate (ASMR) for falls among older adults have been rising, with the ASIR increasing from 1972.47/100,000 in 1990 to 3404.53/100,000 in 2019, reflecting an average annual growth rate of 1.929%. Similarly, the ASMR rose from 20.17/100,000 to 30.67/100,000, with an average annual growth rate of 1.535% [6].

Falls not only inflict physical and psychological harm on patients but also contribute to extended hospital stays and potential medical disputes [7,8]. The financial burden of treating fall-related injuries escalates with aging populations and rising medical expenses [9]. In 2015, the medical expenses associated with falls among older adults amounted to approximately $50 billion in the U.S., exceeded 470 million pounds in the U.K., and averaged over $6,669 per hospitalization in Australia [2]. In China, the cost per fall injury ranged from $16 to $3,812/ person [10]. In response, the WHO has issued guidelines emphasizing the need for healthcare organizations to implement patient safety management systems to prevent adverse events such as falls. The Joint Commission International (JCI) includes fall incidence as a criterion for accreditation [11]. Moreover, China has incorporated fall prevention into its patient safety objectives, as outlined in the “Healthy China 2030” Plan, highlighting the importance of fall prevention interventions for the elderly as a key component of health promotion [12]. The increasing demand for high-quality healthcare services has underscored the importance of fall risk management as a crucial aspect of quality improvement and patient safety in healthcare organizations.

Despite efforts to enhance fall risk management, progress has been hindered by medical staff’ insufficient knowledge, beliefs, and behaviors [13], as well as poor patient compliance [14,15]. As evidenced by nurse-led fall prevention protocols, the primary responsibility for managing fall risk typically lies with nursing staff, with physicians providing input primarily in complex cases [16–18]. This lack of collaboration, coupled with infrequent invitations from nurses for physicians to participate, has resulted in underutilization of physicians’ roles. A study published in The Lancet [19]demonstrated that fall risk management involving physicians, nurses, and patients reduced fall incidence and injury rates compared to conventional management, with fall rates of 7.80/1,000 in the experimental group versus 13.78/1,000 in the control group, and injury rates of 2.63/1,000 versus 4.75/1,000, respectively. A hospital in China implemented a “nurse-led, multi-departmental collaboration model” to prevent falls, resulting in a decrease in fall incidence from 0.07/1,000 to 0.04/1,000 and a reduction in injury rates from 11.54% to 5.0% within one year [20]. These findings highlight the critical role of both medical staff and patient self-management in fall prevention, emphasizing the need to enhance medical staff’ participation and improve patient adherence.

The collaborative role of medical staff and patients in managing fall risk has garnered increasing attention. However, most existing studies focus on either medical staff or patients, with limited systematic analysis of their interactions. This study aims to fill this gap by using a questionnaire survey to explore the attitudes, behaviors, and influencing factors of medical staff and patients in fall risk management. The goal is to provide a scientific basis for developing more personalized, targeted, and effective fall risk management strategies.

## 2. Methods

### 2.1. Design and study population

This study employed a multicenter cross-sectional survey, selecting physicians, nurses, and patients from several provinces across the China using convenience sampling from April 11 2024 to May 31 2024. While this non‑probability method may limit the generalizability of the results, it was chosen for two primary reasons. First, the study is exploratory, aiming to preliminarily assess attitudes and behaviors toward fall risk management among physicians, nurses, and patients. Second, randomized probability sampling was not feasible within the clinical workflow and time constraints of the project. To enhance internal validity, strict inclusion and exclusion criteria were applied to ensure data quality and comparability within the sample.

Inclusion criteria for physicians and nurses included: (1) engagement in clinical work, including those in hospital management positions who also participate in frontline clinical work, (2) possession of a doctor’s qualification certificate or a doctor’s assistant qualification for physicians, and a nurse’s qualification for nurses. Exclusion criteria included those not currently in practice, such as individuals on long-term leave or retired. Inclusion criteria for patients were: (1) age ≥ 18 years, (2) possessed normal communication and literacy abilities, could fill in questionnaires, (3) gave informed consent for voluntary participation in the study. Exclusion criteria for patients included those with critical or deteriorating conditions.

### 2.2. Sample

The target sample size was determined in accordance with established best practices to ensure the robustness of the psychometric analysis [21]. A larger sample reduces measurement error, yields more stable factor loadings, and enhances the reproducibility and generalizability of the results. For scale development of this kind, a widely cited heuristic recommends including 5–10 respondents per questionnaire item [22]. Accordingly, the sample size for this study was calculated using the standard formula for survey research: N=n×(5~10), where ‘n’ represents the number of items [23]. Each version of the questionnaire (for physicians, nurses, and patients) contained 27 items. To account for potential invalid responses, the calculated sample was increased by 20% using the adjustment formula:N=n×(5~10)÷(1−0.20). Based on this calculation, the theoretical sample size range for each group was 169–338 participants. To further ensure the stability and reliability of the findings, we deliberately recruited a sample size exceeding the upper bound of this range for each group.

### 2.3. Data collection

The research team collaborated with administrative offices at participating hospitals. Before distributing the questionnaires, the research team obtained approval from hospital presidents or nursing directors. Following this, department heads or head nurses were responsible for disseminating the questionnaires within their respective WeChat (version8.0.59, https://weixin.qq.com/) groups. Upon accessing the link, participants encountered an introductory page detailing the study purpose, confidentiality protocols, and electronic consent requirements. Only individuals confirming informed consent were directed to the main questionnaire. The distribution and collection of the questionnaires were managed by the Questionnaire Star (https://www.wjx.cn/), which ensured that each IP address could submit only one response. This measure was implemented to maintain data quality and prevent duplicate entries.

### 2.4. Measurements

The research team developed a questionnaire using a literature review and expert consultation method. This included socio-demographic questionnaire and the “questionnaire on attitude and participation in falls risk management from the perspective of physicians, nurses, and patients”. For physicians and nurses, the socio-demographic section collected data on gender, age, marriage, degree of education, hospital level, department of work, years of work experience, professional title, and administrative position. For patients, it was collected socio-demographic such as gender, age, marriage, degree of education, hospital level, department of medicine, occupational status, ways of living and medical payment.

### 2.5. Instrument reliability and validity

Initially, relevant literature was reviewed from both domestic and international databases to construct an initial pool of entries, guided by fall risk management guidelines and expert consensus. The questionnaires for physicians, nurses, and patients each consist of 27 items, comprising both a socio-demographic questionnaire and a fall risk management questionnaire. Subsequently, 11 experts from tertiary hospitals, with an average age of 44.82 ± 6.306 years (range: 35–58) and an average of 20.64 ± 7.827 years of work experience (range: 9–38), participated in two rounds of correspondence. The general characteristics of these experts are detailed in Table 1. Based on their feedback, entries with an importance rating of ≤3 points and a coefficient of variation >0.25 were removed, and necessary modifications and refinements were made. The authority coefficients Ca, Cs and Cr of the questionnaire were all 0.964, and the coefficients of variation for each entry were ≤0.25, indicating high authority and reliability. The *Cronbach’s alpha* coefficients for the physician, nurse, and patient dimensions were 0.976, 0.954, and 0.960, respectively, demonstrating good reliability and validity of the questionnaire.

**Table 1:** Demographic characteristics of the experts (n = 11).

Variables	N(f)	percentage (%)
**Identity**		
Physician	3	27.3
Nurse	8	72.7
**Gender**		
Male	3	27.3
Female	8	72.7
**Age(years)**		
≤ 40	3	27.3
41-50	6	54.5
≥ 51	2	18.2
**Degree of education**		
Undergraduate	2	18.2
Master’s	4	36.4
Doctorate	5	45.5
**Years of working**		
≤ 10	1	9.1
11-20	4	36.4
≥ 21	6	54.5
**Professional title**		
Deputy senior staff	7	63.6
Senior staff	4	36.4

### 2.6. Contents of the questionnaire

The questionnaire was designed to gather comprehensive information from physicians, nurses, and patients regarding fall risk management. The self-assessment component for physicians focused on three primary dimensions: fall risk assessment, fall prevention intervention, and post-fall management. Similarly, the nurse assessment dimension mirrored the structure of the physician section. This section comprised 12 items, each rated on a 5-point Likert scale. Ranging from “I do not agree completely” to “I agree completely”, importance was rated from “very unimportant” to “very important” and implementation from “never” to “always” with scores ranging from 1 to 5 points. The total possible score ranged from 12 to 60 (see Table 2), with higher scores indicating greater participation.

**Table 2:** The description of the questionnaire’s content and scoring.

Groups	Assessment Dimensions	Number of Items	Scoring Criteria	Score Ranges
Physicians	Fall risk assessment	12 items	5-point Likert scale	12 - 60 points
	Fall prevention intervention			
	Post-fall management			
Nurses	Fall risk assessment	12 items	5-point Likert scale	12 - 60 points
	Fall prevention intervention			
	Post-fall management			
Patients	Evaluation of physicians’ participation	8 items	5-point Likert scale	8 - 40 points
	Evaluation of nurses’ participation			
	self-reported adherence			

For patients, it primarily assessed patients’ evaluations of physicians’ and nurses’ participation in fall risk management and their own adherence. This section included 8 items, with scores ranging from 8 to 40 (see Table 2), where higher scores indicated better evaluation and adherence.

The final number of items for the socio-demographic questionnaire combined with the fall risk management questionnaire in each version of the questionnaire (physician, nurse, patient) after the two rounds of expert review, confirming it remained at 27.

### 2.7. Ethical and research approvals

The clinical trial and biomedical ethics committee of West China Hospital of Sichuan University approved the study (No. 2024 [656]). All procedures strictly adhered to the Declaration of Helsinki and relevant regulatory guidelines. Electronic informed consent was obtained from all participating physicians, nurses, and patients following comprehensive explanation of study protocols. Their personal information was strictly protected.

### 2.8. Statistical analysis

Data were meticulously entered into Excel by two specialists from the research team, and statistical analyses were conducted using IBM SPSS software (version 26.0, https://www.ibm.com/). The analysis included:(1) Statistical description, where measurement data were expressed as M ± SD and count data as frequency and composition ratio (%). (2) *Wilcoxon rank-sum test* to compare self-assessed participation by physicians and nurse-assessed participation; paired *t-test* to evaluate differences in patient assessments of physicians and nurses. (3) Difference-Analysis, employing *t-test* or *ANOVA* to compare sample means between groups. (4) Multi-factorial analysis, variables demonstrating statistically significant results in the univariate analysis were subjected to collinearity diagnosis, and those with a variance inflation factor (VIF) <5 were subsequently included in the multivariate analysis. Using linear regression to explore differences in participation of physicians based on varying characteristics. considering a statistical significance level of *P* < 0.05.

## 3. Results

### 3.1. Description of the participants

The study encompassed participants from 19 provinces and cities in China. As a result, 4,272questionnaires were finally analyzed, including 580 from physicians, 2,775 from nurses, and 917 from patients. A description of the participants’ demographic and developmental characteristics is presented in Table 3.

**Table 3:** Demographic and developmental characteristics of the participants.

	Physicians (n = 580)		Nurses (n = 2,775)		Patients (n = 917)		
Variables	N(f)	(%)	N(f)	(%)	Variables	N(f)	(%)
**Gender**					**Gender**		
Male	263	45.3	87	3.1	Male	509	55.5
Female	317	54.7	2688	96.9	Female	408	44.5
**Age(years)**					**Age(years)**		
≤ 29	105	18.1	943	34.0	≤ 44	233	25.4
30 ~ 39	281	48.4	1263	45.5	45-59	326	35.6
≥ 40	194	33.4	569	20.5	≥ 60	358	39.0
**Marriage**					**Marriage**		
Unmarried	125	21.6	741	26.5	Unmarried	89	9.7
Married	446	76.9	1983	71.5	Married	762	83.1
Divorcee	7	1.2	49	1.8	Divorcee	33	3.6
Widowed	2	0.3	2	0.1	Widowed	33	3.6
**Degree of education**					**Degree of education**		
College and below	19	3.3	555	20.0	Primary and below	237	25.8
Undergraduate	253	43.6	2139	77.1	Junior high school	283	30.9
Master’s degree or above	308	53.1	81	2.9	High school	168	18.3
					College and above	229	25.0
**Hospital level**					**Hospital level**		
Second-class hospital and below	35	6.0	190	6.8	Second-class hospital and below	121	13.2
Tertiary hospital	545	94.0	2585	93.2	Tertiary hospital	796	86.8
**Department of work**					**Department of medicine**		
Internal medicine	125	21.5	651	23.5	Internal medicine	296	32.3
Surgery	94	16.2	461	16.5	Surgery	119	13.0
Acute and critical care department	28	4.8	227	8.2	Acute and critical care department	21	2.3
Gynecology and pediatrics	63	10.9	222	8.0	Gynecology and pediatrics	37	4.0
Oncology	171	29.5	734	26.5	Oncology	406	44.3
Geriatrics	18	3.1	72	2.6	Geriatrics	14	1.5
Else	81	14.0	408	14.7	Else	24	2.6
**Years of work experience(years)**					**Occupational status**		
1 ~ 2	90	15.5	300	10.8	In-service	239	26.1
3 ~ 5	106	18.3	468	16.9	Non-employment	489	53.3
6 −10	127	21.9	600	21.6	Else	189	20.6
11-20	160	27.6	1015	36.6	**Ways of living**		
≥ 21	97	16.7	392	14.1	Live alone	78	8.5
**Professional title**					Husband and wife living together	437	47.7
Junior level	194	33.5	1485	53.5	Living with children	173	18.9
Middle level	224	38.6	1089	39.3	Living with parents	65	7.1
High level	162	27.9	201	7.2	Living with partner, parents, children	149	16.2
**Work position**					Else	15	1.6
Resident physician/ Charge nurse	367	63.3	1780	64.2	**Medical payment**		
Medical Team Leader/ Nurse team leader	123	21.2	327	11.8	Self-funded	70	7.6
Instructor/ Head nurse	18	3.1	342	12.3	Rural cooperative medical care	307	33.5
Else	72	12.4	326	11.7	Employee medical insurance	519	56.6
**Administrative position**					Else	21	2.3
No	503	86.7	2411	86.8	**Whose advice would you rather follow about falls risk management**		
Yes	77	13.3	364	13.2	Physicians	518	56.5
					Nurses	353	38.5
					Others	46	5.0

The majority of the medical staff respondents were female (3355, 89.57%). A comparable proportion of physicians (48.4%) and nurses (45.5%) were categorized as young and middle-aged. The majority of both groups were married-76.9% physicians and 71.5% nurses. In terms of education, 53.1% of physicians held a master’s degree or higher, while 77.1% of nurses had undergraduate degrees. The majority of the staff were employed in tertiary hospitals (94.0% physicians vs. 93.2% nurses respectively). A significant portion of physicians (29.5%) and nurses (26.5%) were specifically working in oncology wards. The most common range of work experience for both physicians and nurses were 11–20 years (27.6% vs 36.6%). Over a half of nurses (n = 1485, 53.5%) were junior level, while among physicians, 38.6% (n = 224) were at the middle level. A similar percentage of physicians and nurses were work position (63.3% resident physician vs 64.2 charge nurse). In both professional groups, the majority of staff did not hold any administrative positions.

In our patient cohort, represented age group was individuals over 60 (n = 385, 39.0%). The majority of these patients were married. In terms of education, 30.9% had completed only junior high school. An overwhelming 86.8% were treated in tertiary hospitals, with a notable concentration in oncology wards. Over half (n = 489, 53.5%) were unemployed. Nearly half, at 47.7%, lived with their spouse. The majority of patients relied on employee insurance to cover their medical costs. A significant proportion, 56.5% indicated they would follow their physicians’ advice.

**Fig 1:**
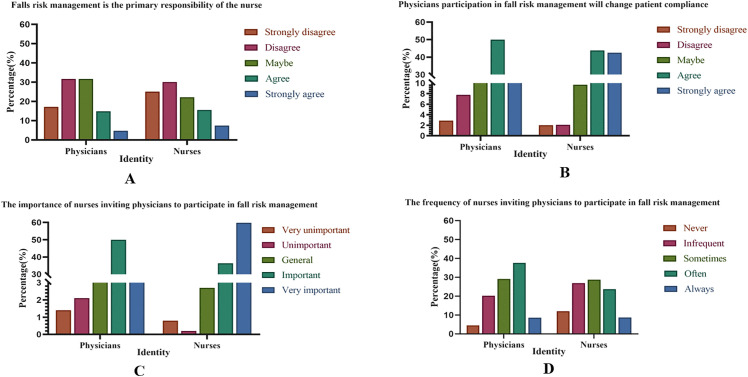
Physicians’ and nurses’ attitudes towards fall risk management.

Fig 1 revealed that nearly half of the physicians (44.8%) and nurses (55.0%) disagreed with the notion that fall risk management is primarily a nurse’s responsibility. A significant majority of physicians (63.8%) and nurses (86.3%) held a positive view that physician involvement enhances patient compliance in fall risk management. Furthermore, 80.0% of physicians and 96.2% of nurses considered it crucial for nurses to invite physicians to participate in managing patient fall risks. However, it was noteworthy that 53.8% of physicians and 67.6% of nurses reported that nurses seldom actively invite physicians to engage in fall risk management.

### 3.2. Comparison of attitudes and participation of medical staff

**Fig 2:**
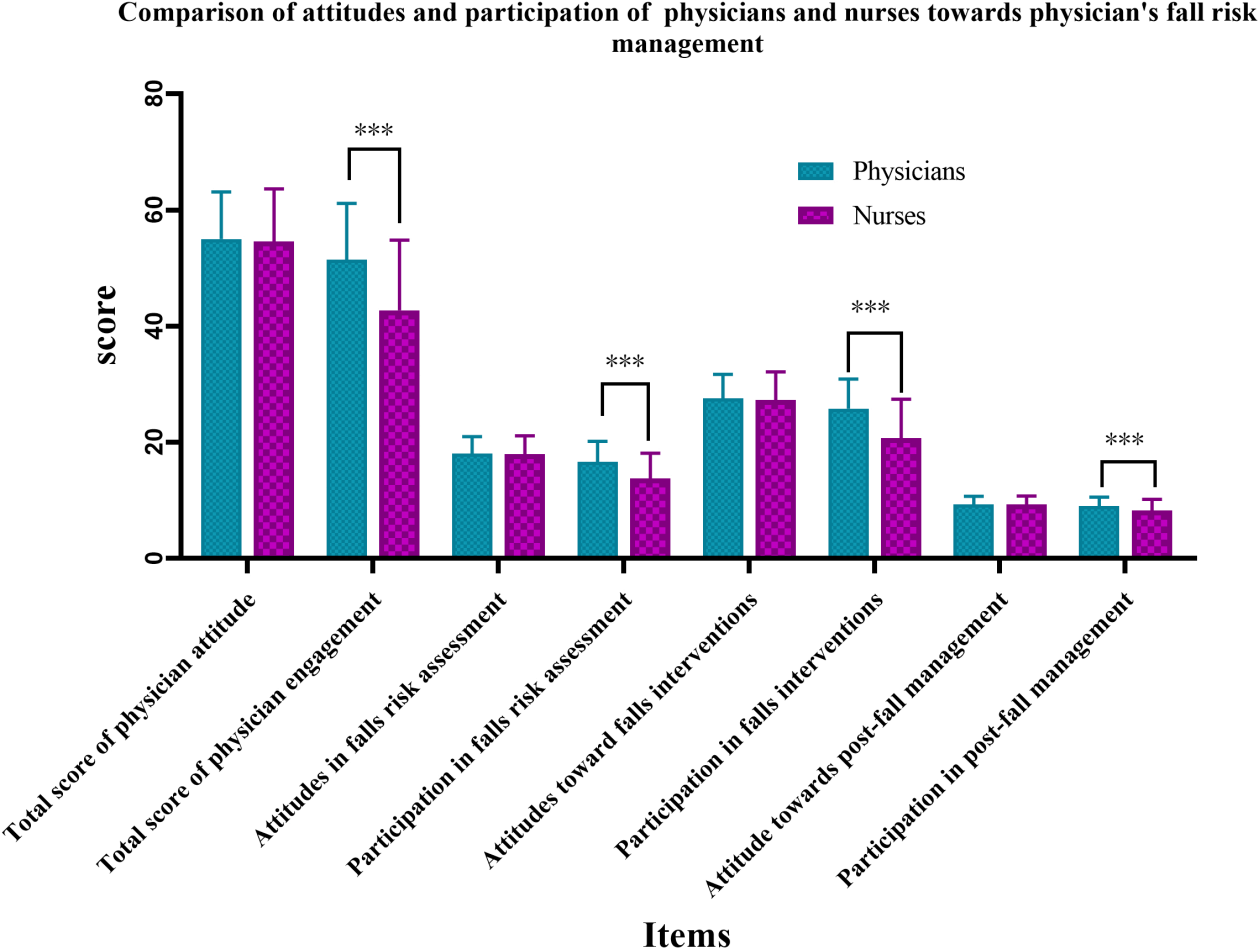
Comparison of attitudes and participation of medical staff ‘self-evaluation & other evaluation’ towards doctor’s fall risk management.

Physicians’ self-assessed scores for fall risk management attitude and participation were 55.02 ± 8.124 and 51.47 ± 9.703, respectively. In contrast, nurses assessed physicians’ fall risk management attitude and participation with scores of 54.58 ± 9.096 and 42.77 ± 12.052, respectively. Upon further examination (Fig 2), the *t-test* for physicians’ attitude scores yielded a *P-value* >0.05, we performed a post*-*hoc power analysis based on the two-sample mean difference test was conducted given the current sample size. The resulting power was 0.21. This suggests that the lack of significant inter*-*group differences may be attributed to insufficient sample size. Future studies should consider expanding the sample size for further investigation. However, a statistically significant difference was observed in the scores related to the implementation of fall risk management (*P* < 0.001), indicating potential deficiencies in physicians’ execution of these practices in clinical work.

### 3.3. Influence factors of physicians’ participation in fall risk management

The linear regression analysis results, as presented in Table 4, indicated several key factors influencing physicians’ participation in fall risk management. Notably, degree of education, gender, the perceived importance of nurses inviting physicians, and the frequency of invitations emerged as significant determinants. Specifically, a higher literacy level among physicians was associated with lower participation in fall risk management. In contrast, female physicians demonstrated higher participation rates compared to their male counterparts. Furthermore, the perceived importance of nurses’ invitations significantly correlated with increased physicians participation. Additionally, a higher frequency of invitations from nurses was linked to greater physicians involvement in fall risk management.

**Table 4:** Multiple linear regression analysis of general information on physicians’ participation in fall risk management.

Physicians	Unstandardized coefficient		Standardized coefficient *β*	*t*	*95% Confidence Intervals*	*P* standard error
	*B*	standard error				
Constant	39.809	2.588		15.380	34.725 ~ 44.893	<0.001
Degree of education	−2.124	0.650	−0.123	−3.265	−3.401 ~ −0.846	0.001
Females	1.739	0.734	0.089	2.369	0.297 ~ 3.181	0.018
the importance of nurses inviting you to falls risk management	1.376	0.480	0.116	2.864	0.432 ~ 2.319	0.004
Frequency of nurses inviting you to participate in falls risk management	3.207	0.386	0.337	8.300	2.448 ~ 3.966	<0.001

### 3.4. Patients’ perception of medical staffs’ participating in fall risk management

The results of this study showed that the patient-rated physician fall risk management participation score averaged 26.21 ± 7.842, while the adherence score was 8.04 ± 2.076. In comparison, the patient-rated nurse fall risk management participation score was 23.84 ± 6.025, with an adherence score was 8.35 ± 1.856. Comparative analyses revealed statistically significant differences between the scores for physician and nurse participation in fall risk management, as well as in patient adherence scores. These results are detailed in Table 5.

**Table 5:** Differential analysis of patients’ perception of physicians’ & nurses’ participating in fall risk management.

Item	Physicians M ± SD	Nurses M ± SD	*t*	*P*	*Mean difference*
Patient evaluation participation	26.21 ± 7.842	23.84 ± 6.025	12.155	<0.001	2.374 (1.991-2.757)
Patient adherence	8.04 ± 2.076	8.35 ± 1.856	−5.24	<0.001	−0.305 (−0.420- 0.191)

### 3.5. Major difficult factors of fall risk management

This study’s systematic analysis reveals differing perspectives on the challenges of implementing fall risk management. Physicians and nurses identified the primary obstacles as patient’s disease, insufficient patient attention, and low patient compliance. Conversely, patients attributed the difficulties mainly to their self-illness, Insufficient knowledge for falls, and environmental condition limitations. For further details, referred to Table 6.

**Table 6:** Ranking of factors that make it difficult to implement fall risk management in medical staff and patients.

Physicians	N(%)	Nurses	N(%)	Patients	N(%)
Patient’s disease	306(52.80%)	Patient’s disease	1450(52.30%)	Self-illness	709(77.30%)
Insufficient patient attention	160(27.60%)	Insufficient patient attention	765(27.60%)	Insufficient knowledge for falls	62(6.80%)
Low patient compliance	74(12.80%)	Low patient compliance	450(16.20%)	Limitations of environmental conditions	50(5.50%)
Busy with work and no time	21(3.60%)	Non-principal responsible person	62(2.20%)	Limited access to knowledge for falls	37(4.00%)
Insufficient knowledge for falls	12(2.10%)	Insufficient knowledge for falls	29(1.00%)	Less social support	30(3.30%)
Non-principal responsible person	7(1.20%)	Busy with work and no time	19(0.70%)	Less fall management by physicians	15(1.60%)
/	/	/	/	Less fall management of nurses	14(1.50%)

## 4. Discussion

This study was a multi-center, large-sample survey design, which encompassed various regions and levels of medical institutions in China. This approach enhanced the representativeness and generalizability of the findings, providing a comprehensive reflection of the attitudes and behaviors of medical staff and patients in fall risk management. Furthermore, the study’s design considered the interactions between medical staff and patients, offering a holistic perspective for constructing an improved patient fall risk management system.

This survey revealed that while physicians, nurses, and patients all recognize the importance of fall risk management, actual participation in implementing these measures is suboptimal. Among the 2,775 nurses surveyed, the expected participation of physicians, based on the typical nurse-to-doctor ratio in China, should have been at least 925. However, only 580 physicians actively participated, indicating low motivation among physicians, similar to findings from a study of 60 hospitals in the United States [24]. Another study found that although participants acknowledged the importance of fall prevention, but 90% of them were willing to spend less than five minutes on it [25]. This may be due to some physicians perceiving fall risk management as primarily a nursing responsibility [3], and believing their involvement has minimal impact on patient adherence [26]. Patients, despite expressing willingness to cooperate, often fail to comply with fall prevention measures due to inadequate risk perception and attention. Active patient involvement in fall management is key to reducing fall rates in hospitals and increasing awareness of fall prevention. This success is tied to better self-awareness among patients, specialized care from medical staff, and broad interventions like improved environments, all of which are vital for effective fall prevention in healthcare.

We found that higher literacy levels among physicians correlate with lower engagement in fall risk management. This might result from insufficient targeted education and training [27]. As well as imbalanced work pressure and time allocation [28,29]. Despite physicians were well-educated, they may lack systematic training in fall risk management during their education and clinical practice. The demands of complex disease diagnosis, clinical teaching, and research further pressure physicians to prioritize more urgent tasks, relegating fall management. Additionally, female physicians were found to be more engaged than their male counterparts, possibly due to personality traits and empathy. Female physicians often exhibit greater empathy [30]and sensitivity to risk [31], enabling them to better understand patient conditions and identify potential fall risks, leading to more active participation in fall risk management.

Importantly, the frequency and perceived importance of nurses’ invitations to physicians significantly correlated with physicians’ participation. Nurses’ invitations enhance physicians’ sense of responsibility in patient safety, increasing their awareness and attention to fall risk management. Consequently, frequent invitations help establish a continuous habit of participation. Thus, encouraging nurses to invite physicians more frequently and advocating for physicians’ active participation are crucial for enhancing the effectiveness of fall risk management.

The study found that patients rated physicians’ participation in fall risk management higher than nurses’. This may stem from patients’ traditional cognitive biases, focusing more on disease treatment and overlooking risk prevention. Physicians are traditionally associated with diagnosis and treatment, while nurses are linked to risk prevention, leading to misconceptions about their roles. Interestingly, patients tend to adhere more to nurses due to the emphasis on fall prevention as a quality care indicator [32]. This focus prompts nurses to implement effective measures to reduce falls and injuries, whereas physicians may pay less attention to specific nursing operations related to fall prevention.

Medical staff and patients often have differing perceptions regarding the challenges of fall risk management. From the perspective of medical staff, three primary factors contribute to the difficulty in managing fall risks: insufficient patient attention, patient’s disease, and low patient compliance. Insufficient patient attention is a significant concern, as many patients fail to recognize the seriousness and potential harm of falls. This lack of awareness leads to a disregard for the advice and preventive measures recommended by medical staff, thereby increasing the risk of falls [33,34]. Additionally, patient disease factors play a crucial role. Certain medical conditions, such as neurological disorders, can impair physical functions, leading to balance disorders [35]and muscle weakness [36]. These conditions complicate the task of accurately predicting and preventing falls, as medical staff must consider the diverse characteristics and effects of various diseases. Moreover, low patient compliance further exacerbates the issue [37]. Despite medical staff dedicating substantial time and effort to developing detailed fall prevention plans and explaining their importance, some patients’ noncompliance hinders the effective implementation of these measures, significantly reducing management effectiveness.

Conversely, patients identify their self-illness, environmental condition limitations, and inadequate knowledge base as the primary challenges in managing fall risks. The presence of diseases such as cardiovascular [38]and skeletal muscle disorders [39]not only restricts mobility but also affects balance and cognitive functions, thereby increasing fall likelihood. Environmental conditions also pose significant challenges [40,41]Factors such as slippery floors, inadequate lighting, and inappropriate furniture layouts can trigger falls, and the inability to promptly modify these conditions to meet individual needs complicates fall risk management. Furthermore, patients often face a lack of knowledge regarding fall prevention [42]. This knowledge gap prevents them from accurately assessing their physical condition and mobility, understanding safe activities, and taking necessary precautions, thereby increasing fall risk and leaving them feeling overwhelmed by fall prevention efforts.

## 5. Limitations

The study had several limitations. First, the use of convenience sampling may restrict the generalizability of the findings, and the cross-sectional design precludes any analysis of temporal changes in attitudes or behaviors. Future research could employ probability sampling and longitudinal designs to enhance representativeness and trace developmental trajectories. Second, the reliance on self-reported quantitative data introduces potential discrepancies between stated attitudes and actual behaviors, and may not fully capture nuanced subjective experiences. Integrating qualitative methods in future studies could help explore underlying influencing factors in greater depth. Third, social desirability bias may have inclined participants to provide responses perceived as socially acceptable, rather than reflecting their true perspectives. Finally, the study’s focus was limited to in-hospital settings, leaving fall risk management in post-discharge contexts unexamined. Subsequent research should broaden its scope and increase the sample size to include transitional and community-based care.

## 6. Implications for practice

In this study, we present an interesting point, to enhance fall risk management, it is crucial to increase nurses’ awareness of the need to involve physicians, encourage nurses to invite physicians more frequently, and motivate physicians to participate actively. These measures are gaining popularity as effective strategies for improving management efficiency.

## 7. Conclusion

This study revealed that physicians, nurses, and patients generally possess a high level of cognitive awareness regarding fall risk management. This shared understanding underscores the collective recognition of the importance of patient safety and establishes a solid foundation for developing an effective fall risk management system. However, a notable discrepancy exists between this high level of cognition and actual participation, particularly among physicians. This gap might be attributed to factors such as physicians’ busy schedules and insufficient knowledge, as well as patients’ limited cognitive abilities and weak self-management skills. To address this issue, medical institutions should develop a comprehensive and systematic fall risk management system. This system should include measures to enhance communication and collaboration between physicians and patients, optimize healthcare resource allocation, and improve professional training programs. Additionally, medical staff should consider patients’ individual differences and provide comprehensive support and guidance to help them overcome self-management barriers, ultimately minimizing fall occurrences and ensuring patient safety and health.

## Supporting information

S1 Fig Description of fall risk management by physicians and nurses. (TIF)

S2 Fig Comparison of attitudes and participation of physicians and nurses. (TIF)

S1 File Data on patients. (XLSX)

S2 File Data on physicians and nurses. (XLSX)

## References

[1] Lee D-CA, Day L, Hill K, Clemson L, McDermott F, Haines TP. What factors influence older adults to discuss falls with their health-care providers?. Health Expect. 2015;18(5):1593–609. doi: 10.1111/hex.12149 26039594 PMC5060806

[2] Ghosh M, O’Connell B, Afrifa-Yamoah E, Kitchen S, Coventry L. A retrospective cohort study of factors associated with severity of falls in hospital patients. Sci Rep. 2022;12(1):12266. doi: 10.1038/s41598-022-16403-z 35851400 PMC9293967

[3] Su Q, Song M, Mao Y, Ku H, Gao Y, Pi H. An analysis of the associated factors for falls, recurrent falls, and fall-related injuries among the older adults in senior Chinese apartments: A cross-sectional study. Geriatr Nurs. 2023;52:127–32. doi: 10.1016/j.gerinurse.2023.05.016 37290218

[4] Moreland B, Kakara R, Henry A. Trends in nonfatal falls and fall-related injuries among adults aged ≥65 years - United States, 2012-2018. MMWR Morb Mortal Wkly Rep. 2020;69(27):875–81. doi: 10.15585/mmwr.mm6927a5 32644982 PMC7732363

[5] Salari N, Darvishi N, Ahmadipanah M, Shohaimi S, Mohammadi M. Global prevalence of falls in the older adults: A comprehensive systematic review and meta-analysis. J Orthop Surg Res. 2022;17(1):334. doi: 10.1186/s13018-022-03222-1 35765037 PMC9238111

[6] Wang YLL. Trends and age-period-cohort model analysis of incidence and mortality of falls among elderly in China from 1990 to 2019. Chin J Evid-Based Med. 2024;24(07):783–91.

[7] Takase M, Kisanuki N, Nakayoshi Y, Uemura C, Sato Y, Yamamoto M. Exploring nurses’ clinical judgment concerning the relative importance of fall risk factors: A mixed method approach using the Q Methodology. Int J Nurs Stud. 2024;153:104720. doi: 10.1016/j.ijnurstu.2024.104720 38408403

[8] Guirguis-Blake JM, Perdue LA, Coppola EL, Bean SI. Interventions to prevent falls in older adults: Updated evidence report and systematic review for the US preventive services task force. JAMA. 2024;332(1):58–69. doi: 10.1001/jama.2024.4166 38833257 PMC13552531

[9] Dormosh N, Abu-Hanna A, Calixto I, Schut MC, Heymans MW, van der Velde N. Topic evolution before fall incidents in new fallers through natural language processing of general practitioners’ clinical notes. Age Ageing. 2024;53(2):afae016. doi: 10.1093/ageing/afae016 38364820 PMC10939375

[10] Peng K, Tian M, Andersen M, Zhang J, Liu Y, Wang Q, et al. Incidence, risk factors and economic burden of fall-related injuries in older Chinese people: A systematic review. Inj Prev. 2019;25(1):4–12. doi: 10.1136/injuryprev-2018-042982 30670560

[11] Merrett A, Thomas P, Stephens A, Moghabghab R, Gruneir M. A collaborative approach to fall prevention. Can Nurse. 2011;107(8):24–9. 22128708

[12] Printing and distributing “the Outline of Healthy China 2030”. People’s Daily. 2016.

[13] Albasha N, McCullagh R, Cornally N, Timmons S. Staff knowledge, attitudes and confidence levels for fall preventions in older person long-term care facilities: A cross-sectional study. BMC Geriatr. 2023;23(1):595. doi: 10.1186/s12877-023-04323-0 37749541 PMC10521420

[14] Bamgbade S, Dearmon V. Fall prevention for older adults receiving home healthcare. Home Healthc Now. 2016;34(2):68–75. doi: 10.1097/NHH.0000000000000333 26835805

[15] Montero-Odasso M, van der Velde N, Martin FC, Petrovic M, Tan MP, Ryg J, et al. World guidelines for falls prevention and management for older adults: a global initiative. Age Ageing. 2022;51(9):afac205. doi: 10.1093/ageing/afac205 36178003 PMC9523684

[16] Runkel KM, Rdesinski RE, Miura LN. Hospitalist perceptions of fall prevention: A comparison of two health care systems. Am J Med Qual. 2021;36(1):36–41. doi: 10.1177/1062860620917206 32383632

[17] Liu J, Deng Z, Zhao L, Cheng Y. Qualitative study of nurses applying the Clinical Practice Guidelines for Fall Prevention in Hospitalized Patients in clinical practice. J Nurs Train. 2023;38(12):1118–22. doi: 10.16821/j.cnki.hsjx.2023.25.008

[18] Morris ME, Webster K, Jones C, Hill A-M, Haines T, McPhail S, et al. Interventions to reduce falls in hospitals: A systematic review and meta-analysis. Age Ageing. 2022;51(5):afac077. doi: 10.1093/ageing/afac077 35524748 PMC9078046

[19] Hill A-M, McPhail SM, Waldron N, Etherton-Beer C, Ingram K, Flicker L, et al. Fall rates in hospital rehabilitation units after individualised patient and staff education programmes: A pragmatic, stepped-wedge, cluster-randomised controlled trial. Lancet. 2015;385(9987):2592–9. doi: 10.1016/S0140-6736(14)61945-0 25865864

[20] Liu WA, Jiang G, Wen Q. The role of multidisciplinary team cooperation model in reducing the incidence of falls in hospitalized patients. Nursing Prac Res. 2020;17(23):127–9.

[21] Boateng GO, Neilands TB, Frongillo EA, Melgar-Quinonez HR, Young SL. Best practices for developing and validating scales for health, social, and behavioral research: A primer. Front Public Health. 2018;6:149. doi: 10.3389/fpubh.2018.00149 29942800 PMC6004510

[22] Tinsley HEA, Tinsley DJ. Uses of factor analysis in counseling psychology research. Journal of Counseling Psychology. 1987;34(4):414–24. doi: 10.1037/0022-0167.34.4.414

[23] Aihemaitijiang S, Ye C, Halimulati M, Huang X, Wang R, Zhang Z. Development and validation of nutrition literacy questionnaire for the chinese elderly. Nutrients. 2022;14(5):1005. doi: 10.3390/nu14051005 35267979 PMC8912634

[24] Turner K, Staggs V, Potter C, Cramer E, Shorr R, Mion LC. Fall prevention implementation strategies in use at 60 United States hospitals: A descriptive study. BMJ Qual Saf. 2020;29(12):1000–7. doi: 10.1136/bmjqs-2019-010642 32188712 PMC7501087

[25] Davenport K, Cameron A, Samson M, Sri-On J, Liu SW. Fall Prevention knowledge, attitudes, and behaviors: A survey of emergency providers. West J Emerg Med. 2020;21(4):826–30. doi: 10.5811/westjem.2020.4.43387 32726252 PMC7390582

[26] Meekes WMA, Leemrijse CJ, Weesie YM, van de Goor IAM, Donker GA, Korevaar JC. Falls prevention at GP practices: A description of daily practice. BMC Fam Pract. 2021;22(1):190. doi: 10.1186/s12875-021-01540-7 34548022 PMC8454103

[27] Chen Q, Li M, Wu N, Peng X, Tang G, Cheng H, et al. A survey of resident physicians’ perceptions of competency-based education in standardized resident training in China: A preliminary study. BMC Med Educ. 2022;22(1):801. doi: 10.1186/s12909-022-03863-0 36397045 PMC9673373

[28] Liu Y, Tan Tj, Ngwayi JRM, Zhuang X, Ding Z, Chen Y, et al. Work Patterns and Intensity of Chinese Surgical Residents- A Multicenter Time-and-Motion Study. J Surg Educ. 2024;81(1):76–83. doi: 10.1016/j.jsurg.2023.09.005 37852874

[29] Nguyen MT, Honcharov V, Ballard D, Satterwhite S, McDermott AM, Sarkar U. Primary care physicians’ experiences with and adaptations to time constraints. JAMA Netw Open. 2024;7(4):e248827. doi: 10.1001/jamanetworkopen.2024.8827 38687477 PMC11061766

[30] Pavlova A, Wang CXY, Boggiss AL, O’Callaghan A, Consedine NS. Predictors of physician compassion, empathy, and related constructs: A systematic review. J Gen Intern Med. 2022;37(4):900–11. doi: 10.1007/s11606-021-07055-2 34545471 PMC8452146

[31] Zimmer M, Czarniecki DM, Sahm S. Gender-sensitive considerations of prehospital teamwork in critical situations. Philos Ethics Humanit Med. 2024;19(1):3. doi: 10.1186/s13010-024-00153-z 38504354 PMC10953181

[32] Bernet NS, Everink IH, Schols JM, Halfens RJ, Richter D, Hahn S. Hospital performance comparison of inpatient fall rates; the impact of risk adjusting for patient-related factors: A multicentre cross-sectional survey. BMC Health Serv Res. 2022;22(1):225. doi: 10.1186/s12913-022-07638-7 35180859 PMC8857794

[33] Dolan H, Slebodnik M, Taylor-Piliae R. Older adults’ perceptions of their fall risk in the hospital: An integrative review. J Clin Nurs. 2022;31(17–18):2418–36. doi: 10.1111/jocn.16125 34786777

[34] Naseri C, McPhail SM, Haines TP, Morris ME, Shorr R, Etherton-Beer C, et al. Perspectives of older adults regarding barriers and enablers to engaging in fall prevention activities after hospital discharge. Health Soc Care Community. 2020;28(5):1710–22. doi: 10.1111/hsc.12996 32337796 PMC7513672

[35] Sullivan R, Harding K, Skinner I, Hemsley B. Falls in patients with communication disability secondary to stroke. Clin Nurs Res. 2023;32(3):478–89. doi: 10.1177/10547738221144214 36541748

[36] Satoh M, Miura T, Shimada T, Hamazaki T. Risk stratification for early and late falls in acute care settings. J Clin Nurs. 2023;32(3–4):494–505. doi: 10.1111/jocn.16267 35224808 PMC10078671

[37] Bergeron CD, Friedman DB, Messias DKH, Spencer SM, Miller SC. Older women’s responses and decisions after a fall: The work of getting “back to normal”. Health Care Women Int. 2016;37(12):1342–56. doi: 10.1080/07399332.2016.1173039 27050835

[38] Denfeld QE, Turrise S, MacLaughlin EJ, Chang P-S, Clair WK, Lewis EF, et al. Preventing and Managing Falls in Adults With Cardiovascular Disease: A Scientific Statement From the American Heart Association. Circ Cardiovasc Qual Outcomes. 2022;15(6):e000108. doi: 10.1161/HCQ.0000000000000108 35587567

[39] Yamada M, Kimura Y, Ishiyama D, Otobe Y, Suzuki M, Koyama S, et al. Combined effect of lower muscle quality and quantity on incident falls and fall-related fractures in community-dwelling older adults: A 3-year follow-up study. Bone. 2022;162:116474. doi: 10.1016/j.bone.2022.116474 35752409

[40] Poh FJX, Shorey S. A literature review of factors influencing injurious falls. Clin Nurs Res. 2020;29(3):141–8. doi: 10.1177/1054773818802187 30227728

[41] Colón-Emeric CS, McDermott CL, Lee DS, Berry SD. Risk assessment and prevention of falls in older community-dwelling adults: A review. JAMA. 2024;331(16):1397–406. doi: 10.1001/jama.2024.1416 38536167 PMC12224174

[42] Jarden RJ, Cherry K, Sparham E, Brockenshire N, Nichols-Boyd M, Burgess S, et al. Inpatients’ experiences of falls: A qualitative meta-synthesis. J Adv Nurs. 2025;81(1):4–19. doi: 10.1111/jan.16244 38808473 PMC11638511

